# A protocol to quantify cross-sectional and longitudinal differences in duction patterns

**DOI:** 10.3389/fnins.2024.1324047

**Published:** 2024-06-11

**Authors:** Kevin T. Willeford, Victoria Copel, Hua Rong

**Affiliations:** ^1^Department of Optometric Sciences, NOVA Southeastern University College of Optometry, Fort Lauderdale, FL, United States; ^2^Tianjin International Joint Research and Development Centre of Ophthalmology and Vision Science, Eye Institute and School of Optometry, Tianjin Medical University Eye Hospital, Tianjin, China

**Keywords:** ocular motility, motility patterns, clinical protocol, strabismus, non-strabismic binocular vision dysfunction

## Abstract

Currently, there is no established system for quantifying patterns of ocular ductions. This poses challenges in tracking the onset and evolution of ocular motility disorders, as current clinical methodologies rely on subjective observations of individual movements. We propose a protocol that integrates image processing, a statistical framework of summary indices, and criteria for evaluating both cross-sectional and longitudinal differences in ductions to address this methodological gap. We demonstrate that our protocol reliably transforms objective estimates of ocular rotations into normative patterns of total movement area and movement symmetry. This is a critical step towards clinical application in which our protocol could first diagnose and then track the progression and resolution of ocular motility disorders over time.

## 1 Introduction

There is currently no system to quantify patterns of ocular ductions. This is because most comparisons to others (cross-sectional) or to oneself (longitudinal) are made via subjective observations and codified with discrete numeric scales (von Noorden, 1996; Borchert, 2005). The absence of objective and continuous measurements leaves clinicians with a low-resolution estimate of their patient’s ocular range of motion. Furthermore, because ductions are typically treated as individual entities, descriptions of the total extent and/or symmetry of the ocular range of motion are sparse (Rowe and Hanif, 2011; Salvi et al., 2015; Campi et al., 2021). This lack of quantifiable patterns and criteria by which to detect their change stands in contrast to other areas of eye care where summary statistics derived from imaging technologies endow clinicians with the ability to gauge whether entire structures [e.g., the cornea (Doctor et al., 2020) and optic nerve (Hwang and Kim, 2012; Sullivan-Mee et al., 2013)] or functions [e.g., the visual field (Flammer, 1986)] display patterns indicative of pathologic progression or therapeutic improvement.

Conventional video-based eye trackers, which use the location of the pupil and corneal reflection to estimate gaze position, fall short in addressing this issue because their recording range is narrower than the full range of ocular motion (Lee et al., 2019). The high spatial and temporal resolution of video-based eye trackers are instead ideal for detecting pathological eye movement dynamics (e.g., velocity) (Wong et al., 2006; Kemanetzoglou et al., 2021). Attaining objective measurements of both dynamic and static (i.e., the range of motion) properties of ocular motor function is ideal because post-insult motor adaptations may normalize eye movement dynamics yet leave the eye’s range of motion into the paretic field limited (Wong et al., 2006). Currently, manual techniques such as the lateral version light reflex test (Urist, 1967) and the limbus test (Kestenbaum, 2013) are used to measure the ocular range of motion in clinic. The basic premise of each approach is to compare the location of the limbus in eccentric gazes to its location in primary gaze: the difference in location is then used to estimate the magnitude and direction of ocular movement. A modified limbus test reliant upon digital photography has been used to measure versions in non-strabismic individuals (Lim et al., 2014a) and in patients with muscle overactions (Lim et al., 2014b), thyroid ophthalmopathy (Leite et al., 2020), and ocular blow-out fractures (Lee et al., 2015). This method was also used to confirm an age-related decline in supraduction (Lee et al., 2019) originally documented with other techniques (Clark and Isenberg, 2001; Shechtman et al., 2005; Kang, 2009). Recently, incorporation of machine learning elements which automatically segment the limbal boundaries has drastically reduced the workload required to derive the movement estimates (Kang et al., 2022; Lou et al., 2022). This rapidity has placed automatic ocular-motor diagnostics closer to a clinical reality; however, there are several important steps to take prior to clinical application. First, because there have been limited attempts to quantify the global patterns of ductions, there is no framework available to judge whether a participant’s movements are overall indicative of pathology. Second, criteria for determining longitudinal changes in ductions and their associated patterns have not yet been established. This is important to address because the high amount of interparticipant variability present in normative ranges may make them less sensitive to change. Summary statistics with both features (i.e., intraparticipant comparisons and global amalgamation of data) have distinct diagnostic advantages because they simultaneously minimize the variance imposed by interparticipant factors (e.g., age, race, gender, and ocular biometrics) and maximize the variance present within or between a participant’s eyes (Sullivan-Mee et al., 2013). Thus, it is now time to leverage the automaticity of machine learning towards development of an ocular motility focused device like those routinely used for pattern and change analyses of the anterior segment (Greenstein et al., 2011), posterior segment (Leung et al., 2010), and visual function (Artes et al., 2011). This is essential to transform qualitative descriptors of both normal (e.g., “each eye’s range of motion is similar”) and abnormal (e.g., “the left lateral rectus is weak”) duction patterns into an intuitive database of biometric statistics.

In this paper, we present a proof of concept for our protocol which combines image processing, a framework of summary indices, and change criteria which are designed to initially describe and then detect both cross-sectional and longitudinal changes in a participant’s pattern of ductions. We first develop the techniques necessary for image analysis, next define mathematics which capture duction extent and symmetry, and last define criteria by which to establish clinically meaningful differences. We set forth a series of benchmarks related to each component to establish the validity of our protocol. The analyses and results described together show that our proposed protocol has the potential to first identify and then track the progression and resolution of ocular motility disorders over time.

## 2 Methods

### 2.1 Participants

Twenty non-strabismic participants (3 male, 17 female) between the ages of 22 and 48 years (95% CI: 25.0 to 27.7 years) were recruited to participate in the study. Each participant reported for a single recording session, lasting approximately 30 min, in which ductions of the right and left eye were recorded. All participants were required to take part in an informed consent discussion, and subsequently provide their consent, prior to beginning the experiment. This included evaluation of exclusion criteria (i.e., the presence of strabismus, amblyopia, and/or other neurological diseases). All experiments were reviewed and approved by Nova Southeastern University’s Institutional Review Board and conformed to the principles and applicable guidelines for the protection of human subjects in biomedical research.

### 2.2 Protocol

#### 2.2.1 Image processing

##### 2.2.1.1 Capture

We used an ELP brand digital camera (Shenzhen Ailipu Technology Company, Shenzhen, China) to capture images of the right and left eye in primary and eccentric gaze positions. Each participant was instructed to first look “straight ahead” and then to sequentially move their eye “as far as possible” along one of four pre-defined meridians. A modified tangent screen, constructed with cords of rope stretched across the horizontal, two diagonal, and vertical meridians, was centered 40 cm in front of each participant and was used to guide fixation (Supplementary Figure S1). Each meridian’s length of rope was constructed such that it extended beyond the mean maximal duction in each direction of gaze (Lim et al., 2014a; Lee et al., 2019). For example, assuming a viewing distance of 40 cm and a maximal duction of 70°, each rope’s length was made to be approximately 220 cm [ $70^{{}^{\circ}}=\mathrm{atan}\left(110/40\right)]$ . Several steps were taken to prepare each participant for recording. First, the non-recorded eye was occluded with an adhesive eyepatch. Next, one drop of Proparacaine HCL 0.5% was instilled in the eye to be recorded. Third, a white and black bullseye sticker was adhered to the lower cheek below the recorded eye. This was done to compensate for minor head translations which occurred during the recording. Fourth, each participant placed their mouth unto a custom bite bar apparatus. The apparatus consisted of the bite bar, a forehead rest and headband which together stabilized each participant’s head. Last, immediately prior to the commencement of recording, an ophthalmic speculum was inserted to stabilize and widen the palpebral aperture. This was done to minimize obscuration of the limbus by the lids. Frame capture, which occurred at 30 frames per second, began once a participant was stably fixating straight ahead. This was accomplished by instructing each participant to look “straight ahead” via fixation of a black knot indicating the center of the tangent screen. Each participant then performed at least five alternating fixations along each meridian (e.g., left, right, left, right, etc). While eccentrically fixating, participants were encouraged to move their eyes “as far as possible” along the rope guides until they could look no further. After a total of at least five alternating fixations were performed, participants then continued onwards to alternately fixate along the next meridian (e.g., up and left, down and right, up and left, down and right, etc.). This was done until ductions had been performed along all four meridians, a process which lasted approximately 1 min. The 30 fps sampling rate captured many frames per fixation; thus, to “distill” the data into one frame per fixation, one of the authors (KW) manually selected a single frame in which the eye had appeared to move the furthest from each fixation’s collection of frames. This resulted in a total of eighty-two frames (two in primary gaze and five fixations per eight ductions in each eye) for each participant.

### 2.3 Segmentation

Benchmark I: model metrics are gaze independent.

Each of the frames was next submitted to a mask recurrent convolutional neural network (R-CNN) which segmented the iris, and thus, demarcated the limbus boundaries. The mask R-CNN is ideal for this task because it performs instance segmentation, a type of image segmentation that demarcates the shapes of objects on a pixel-by-pixel level (He et al., 2017). Our mask R-CNN was built on a ResNet 101 backbone, instantiated in PyTorch, and initially trained on a collection of ground truth images annotated by the co-authors (KW & HR). The ground truth labels were created by first downloading a publicly available dataset of eye images on Kaggle and then using Photoshop to place elliptically shaped masks over visible portions of the iris. For images in which the eyelids obscured portions of the iris, the elliptical masks were chosen to best match the curvature of the visible portions of the iris. Images in which more than half of the iris was obscured were not annotated or used for training. Training was performed by feeding the annotated ground truth images into the mask R-CNN using a stochastic gradient descent (SGD) optimization algorithm. The learning rate was set to 0.001, the momentum to 0.9, and the weight decay to 0.0005. A learning rate scheduler was employed to decay the learning rate by a factor of 0.2 every 10 epochs. A validation set of labeled images captured with the co-authors’ (KW & HR) eyes gazing into various gazes, held open with an ophthalmic speculum, were used to fine tune the model parameters and conclude training. All frames captured from each participant performing ductions were then submitted to the mask R-CNN for iris segmentation. The diameter of each segmented iris was taken to represent the boundaries of the limbus. Supplementary Video S1 shows an example of identified limbus boundaries in one participant.

We evaluated the performance of our mask R-CNN using a test set of sixty randomly selected frames (three from each participant’s right and/or left eyes). The accuracy, sensitivity, specificity, and precision were computed in the following manner (Eqs 1–4):


(1) $$\mathrm{Accuracy}=\frac{TP+TN}{TP+TN+FP+FN}$$

(2) $$\mathrm{Sensitivity}=\frac{TP}{TP+FN}$$

(3) $$\mathrm{Specificity}=\frac{TN}{TN+FP}$$

(4) $$\mathrm{Precision}=\frac{TP}{TP+FP}$$

True positives (TP), true negatives (TN), false positives (FP), and false negatives (FN) represent correctly marked pixels, pixels that were correctly excluded, pixels that were mistakenly marked, and pixels that were incorrectly excluded. Each of the metrics reveals a different aspect of the mask R-CNN’s segmentation performance: accuracy is the ratio of correct predictions compared to all predictions, sensitivity shows the proportion of ground truth labeled pixels that were correctly marked, specificity shows the proportion of non-labeled pixels that were correctly excluded, and precision shows the ratio of correct markings over all markings. We sorted and then compared each metric by and across duction types to determine whether the mask R-CNN was able to segment the iris, and thus identify the limbus, equivalently across all gaze positions.

### 2.4 Optimization

Benchmark II: optimization error is gaze independent.

**Figure 1:**
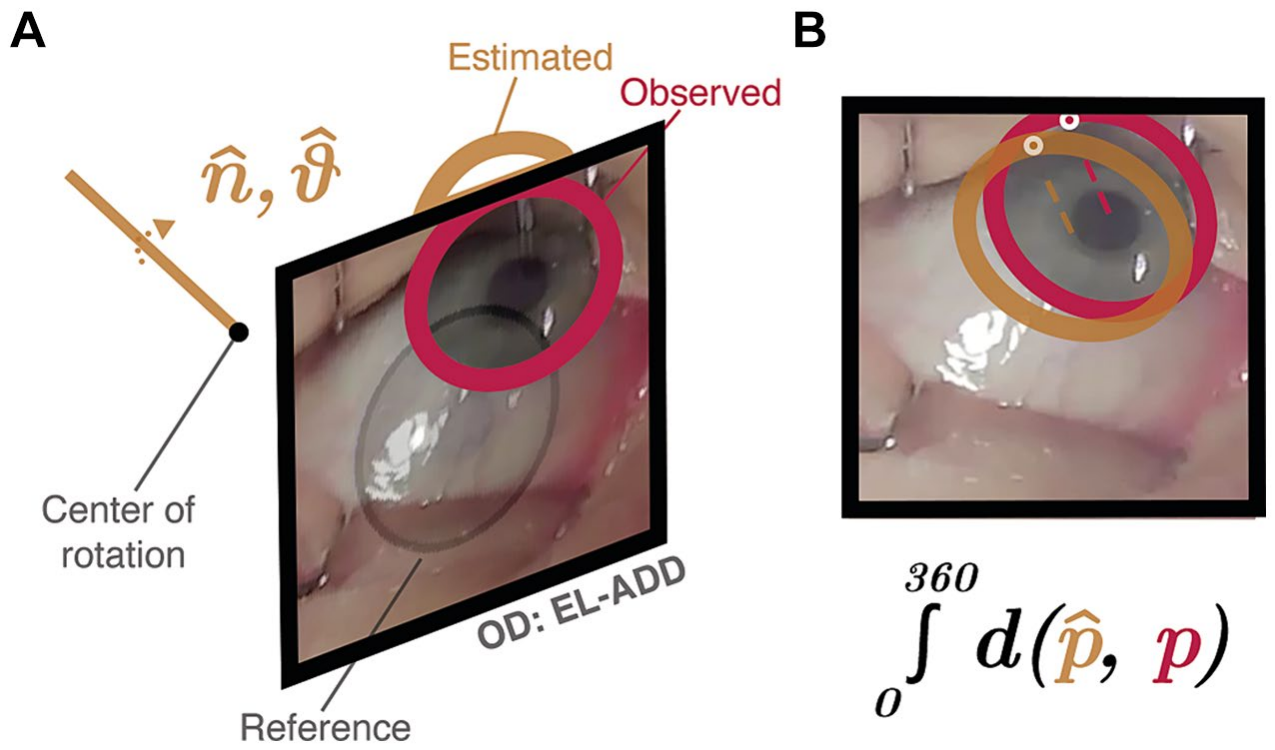
Example of the optimization routine estimating the magnitude and direction of ocular rotation for a duction of the right eye. **(A)** The magnitude (*θ*, angle) and direction (*n*, axis of rotation) of the observed movement (*red*) were estimated by finding the parameters which produced the closest matching 2D projected ellipse. This was done by continually rotating each participant’s reference ellipse (*black*) to a new 3D location (*brown*) and **(B)** minimizing the distance between corresponding points on the estimated ( $\hat{p}$ , *brown*) and observed ( p , *red*) ellipses.

The preceding steps of image capture and segmentation produced eighty-two sets of limbus coordinates in each participant. The basis for our estimation of ocular rotations is a comparison between the limbus coordinates identified in primary vs. eccentric gaze positions. This comparison is an ideal proxy for movement estimation because, unlike the pupil and corneal reflection (Nyström et al., 2016), the physical dimensions of the limbus are stable. Thus, because the apparent location and shape of the limbus change only during ocular rotations, comparison of limbus coordinates can be used to estimate the magnitude and direction of movement (Wang and Sung, 2001; Wang et al., 2005). We used this philosophy to develop an optimization routine whose outputs were the magnitude and direction of ocular rotation. To do this, we considered the boundaries identified in each participant’s primary gaze to represent a reference ellipse from which all eccentric rotations began. The task of the optimization routine was then to find the rotation, which when applied to the reference ellipse, produced an estimated ellipse which best matched the observed elliptical boundaries present in eccentric gaze positions (Figure 1). This strategy removes the need to utilize pre-defined meridians along which to measure movements and is thus capable of estimating the magnitude and direction of any rotation.

We applied several biometric assumptions and unit conversions before beginning optimization. First, the center of rotation was assumed to lie 10.45 mm behind the limbus for all participants. This designation assumes a corneal sagittal depth of 2.5 mm (Sorbara et al., 2013) and an ocular center of rotation located 12.95 mm from the corneal apex. This is a reasonable assumption for all participants given recent evidence that the location of the eye’s center of rotation is independent of the size of one’s globe (i.e., axial length) (Clark and Demer, 2020). The center of rotation is thought to translate during eye movements; however, because the direction and magnitude of such translations is participant-dependent and would require additional free parameters in our optimization routine, we chose to exclude them from our movement estimates and instead assume that the center of rotation remains fixed throughout all ocular rotations. Second, we assumed all axes of rotation were located within a single plane coincident with the center of rotation (i.e., Listing’s Plane). Third, the limbus coordinates were converted from pixels to millimeters to make the units of the limbus boundaries coincident with those of the biometric parameters. For this conversion, we assumed that each participant had a horizontal visible iris diameter of 11.75 mm (Bergmanson and Martinez, 2021). This allowed us to convert each set of limbus coordinates from pixels to millimeters by using the horizontal width of each participant’s reference limbus as the denominator in a conversion factor (i.e., millimeters per pixel = 11.75 mm / horizontal width of reference limbus in pixels). Fourth, image registration was used to determine if head movements had occurred: this was done by using MATLAB’s imregcorr function to compare images of the bullseye sticker captured in the reference primary gaze positions to those of the bullseye sticker captured in eccentric gaze positions. This function was used to identify displacements of the sticker in pixels and has a one-pixel resolution. Each participant’s conversion factor was used to convert the displacements into millimeters and then shift the limbus coordinates opposite the displacements to counteract the intrusion of head movements into the data. Note that this correction can only account for translations, and not rotations, of the head. Given the concurrent use of a bite-bar, we believe that the potential for either head translations and/or rotations to confound our data was minimal. This is shown by the minimal amounts of translation detected by sticker movement: the mean horizontal and vertical displacements were 0.24 and 0.31 mm, respectively. Further, given that *imregcorr’s* one-pixel resolution, equivalent to 0.10 mm at the viewing distance used, was smaller than the displacements observed, image registration could also be used to minimize the impact of head movements in less constrained experimental environments where the amount of head movement is likely to be larger. Supplementary Video S2 shows the outcome of this correction procedure in one participant.

Rotation parameters were next estimated using mathematical optimization. Each observed set of eccentric elliptical limbus boundaries were compared to their respective reference ellipses using an optimization routine was run by MATLAB’s fminsearch . The routine was tasked with estimating three free parameters: the axis ( $n=\left[\mathrm{param}1\;\mathrm{param}2\;0\right]$ ) and angle ( θ=param3 ) of rotation, with param1 and param2 representing the axes about which vertical and horizontal rotations, respectively, occurred. The minimum and maximum parameter values were bounded between −1 and 1 for the axis and between 0 and 90 for the angle. Each iteration of the routine produced an estimated ellipse by applying a rotation to the reference ellipse. The estimated ellipse was then compared to the observed ellipse using an error function which computed the total distance between corresponding points (i.e., those sharing the same angles relative to center) on the estimated ( $\hat{p}$ ) and observed ( $\hat{p}$ ) ellipses (Eq. 5). Optimization using this error function produces ellipses which are most similar in both location and shape.


(5) $$\varepsilon =\overset{360}{\underset{0}{\int }}\mathrm{distance}\left(\hat{p,}p\right)$$

The routine continued until the error between the observed and estimated ellipses was minimized. At this point, the minimum error and optimized parameters for the comparison were saved. The optimized axis and angle for each observed vs. reference comparison were taken to represent the direction and magnitude of each duction, respectively. We last selected three rotation estimates out of the possible five for each duction and participant using a criterion of least error. The least error estimates were chosen within each duction and participant to ensure that each participant’s eight ductions were represented in the group mean data. This resulted in a total of forty-eight estimated rotations (three selected x eight ductions per eye) for each participant. As with the preceding model metrics, we sorted and then compared the mean error values across duction types to determine whether our optimization scheme estimated rotations equivalently across gaze positions.

### 2.5 Pattern quantification

**Figure 2:**
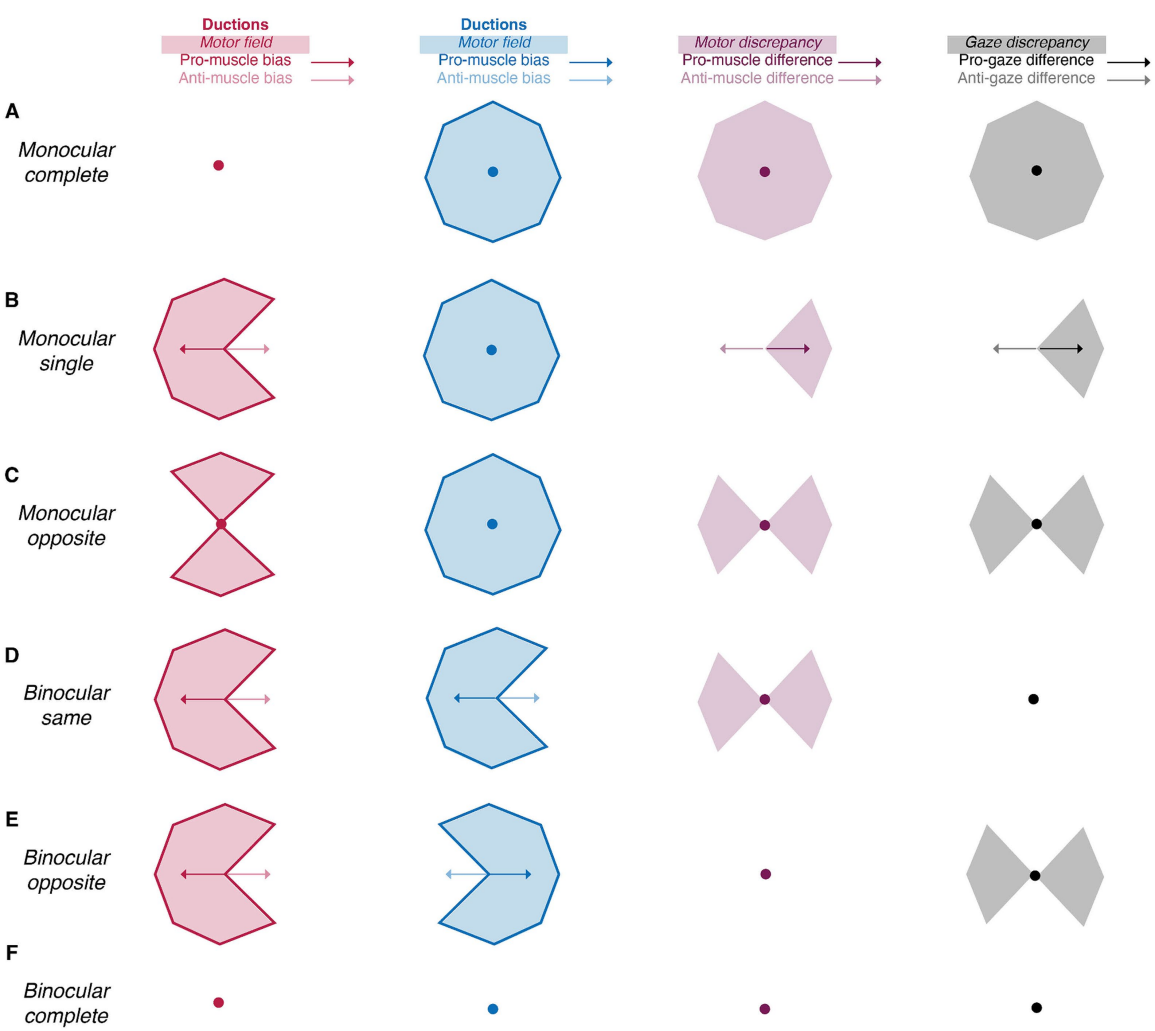
Summary indices reveal the laterality and type of ocular motility disorders **(A-F)**. The amalgamation of area (shaded areas) and symmetry (arrows and circles) indices uniquely characterizes the laterality (monocular vs. binocular) and type (single, synergists, agonists, or complete) of motility defects. Red, blue, purple, and black shaded areas represent the right and left motor fields, the motor discrepancy, and the gaze discrepancy, respectively. Similarly, the red, blue, purple, and black and arrows represent the right and left muscle biases, the muscle difference, and the gaze difference. Arrows represent vectors with non-zero magnitude whereas circles represent vectors with no magnitude.

In the second step of our duction recording protocol, we characterize each participant’s motility pattern using a framework of summary indices (Table 1). This framework is built on the philosophy that all ocular motility defects can be represented by a distinct set of monocular shapes (Figure 2, first two columns). The vertices of each shape are formed by a participant’s ductions. Then, scaling, rotation, or combination of the shapes accounts for differences in the absolute magnitudes and directions of one’s movement limitations. For example, unilateral pareses are described by the “monocular single” set containing a wedge and an octagon (Figure 2B). The size and orientation of the right eye’s wedge, shown in the first column and second row of Figure 2, indicates a complete adduction deficit. Scaling and rotation of this shape can be performed to represent a single duction deficit of any magnitude or direction. The “monocular opposite” set, indicative of movement limitation along an entire meridian, includes an octagon and a bow-tie shape (Figure 2C). As before, the bow tie can be scaled or rotated to represent unilateral horizontal and/or vertical gaze palsies of different magnitudes. The “binocular same” (Figure 2D) and “binocular opposite” (Figure 2E) sets, containing wedges of similar or opposing orientation, are representative of gaze or vergence palsies, respectively. Our framework quantifies each shape by describing its boundaries ( $\vec{d\;}$ , ductions), area ( MF , motor field), and symmetry ( $\vec{MB}$ , muscle bias). This results in a numerical system capable of characterizing any ocular motility disorder. This concept is akin, though not identical, to the use of Zernike polynomials for describing refractive errors. In our framework, we employ simple polygons as opposed to polynomials (Campbell, 2003; McAinden et al., 2011). Note that the number of vertices within each shape represent the number of ductions measured. For cases in which ocular-motor deficits occur symmetrically about a meridian, as in Figure 2B, a higher sampling density (i.e., number of measured ductions) would be redundant because movement ability recovers similarly as gaze is shifted from purely horizontal to horizontal and up and from purely horizontal to horizontal and down. However, in cases where deficits are asymmetric about one or more meridians, a higher sampling density is necessary to veridically “capture” the pattern of motility.

**Table 1:** Framework of summary indices.

*BOUNDARIES*	Magnitude of movement in a single direction
*AREA*	What is the total magnitude of movement across all directions?
Motor fields ( $MF_{OD}$ , $MF_{\mathrm{OS}}$ )	Monocular area enclosed by all ductions
Discrepancies *(MDisc, GDisc)*	Proportion of “monocular only” areas obtained when comparing the motor fields; either matching ductions or gazes compared
*SYMMETRY*	Is movement magnitude the same in all directions?
Muscle biases ( ${\vec{MB}}_{OD}$ , ${\vec{MB}}_{\mathrm{OS}}$ )	Magnitude of monocular asymmetry and direction of smallest / largest ductions
Differences ( $\vec{\mathrm{MDiff}}$ , $\vec{\mathrm{GDiff}}$ )	Magnitude of interocular asymmetry and direction of smallest / largest difference in ductions; either matching ductions or gazes compared

The framework’s indices summarize movement patterns (area and symmetry). Movement patterns provide a global view of intraocular (e.g., muscle biases, motor field) and interocular (e.g., differences and discrepancies) motility which is not always apparent when viewing individual ductions alone.

**Figure 3:**
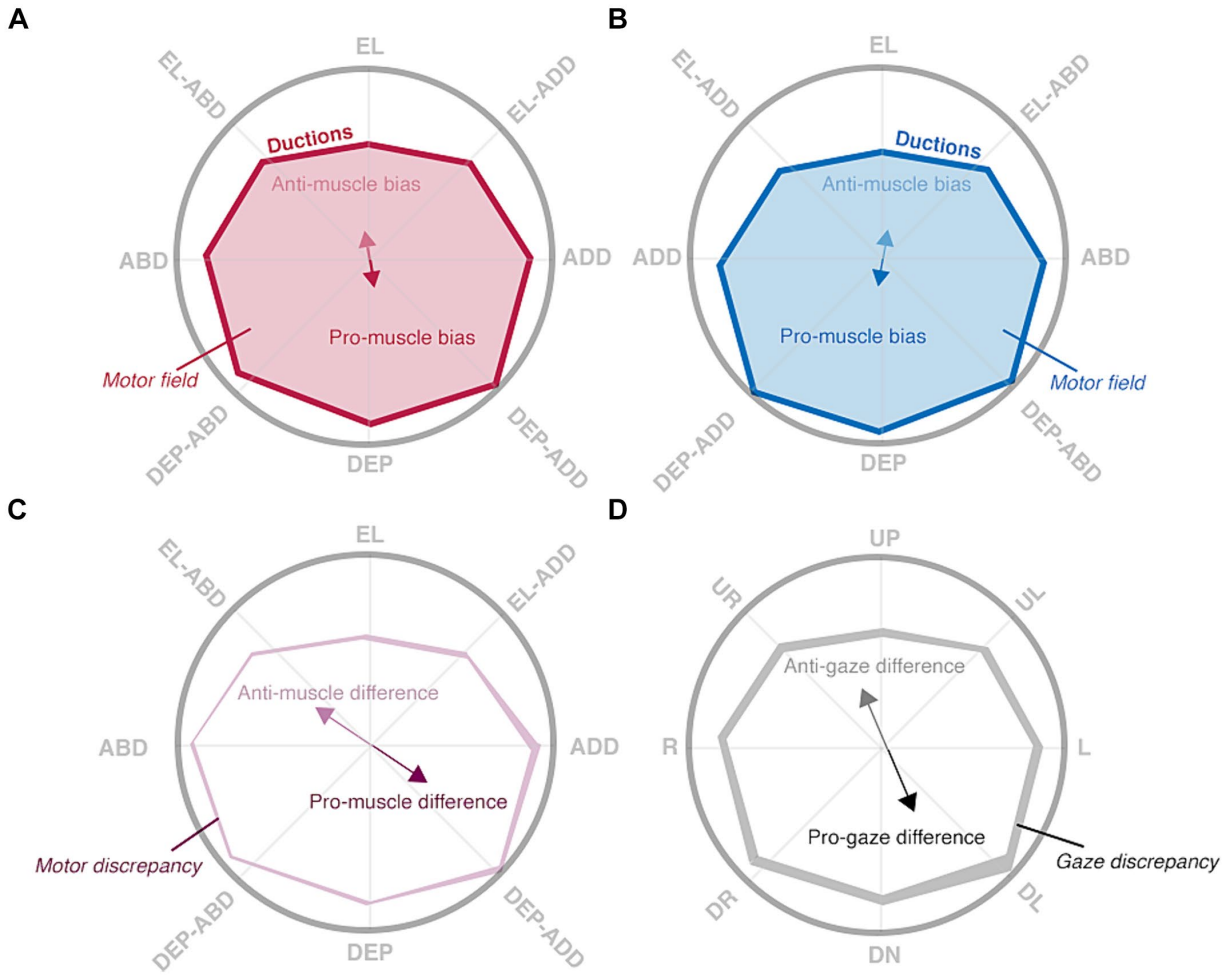
Hypothesized patterns of normal ductions. Ductions are measured by asking a participant to look “as far as possible” in multiple directions, denoted by rope guides, with their right **(A)** and left **(B)** eyes. Ductions (red and blue solid lines) of both eyes are anisotropic: depressive movements are typically largest. This asymmetry is captured by the muscle biases (red and blue solid arrows) and anti-muscle biases (red and blue translucent arrows) which point towards the largest and smallest movements, respectively. The motor fields (red and blue translucent shading) are the areas enclosed by all ductions. Interocular comparisons show the degree of “mismatch” between matching ductions **(C)** or gaze positions **(D)**. The muscle differences (purple arrows) and gaze differences (black) show where matching ductions or matching gazes are most different, whereas the motor discrepancy (translucent purple shading) and gaze discrepancy (translucent black shading) hold the total area of mismatch.

The following sections enumerate the derivation of each index in the framework and show how the differencing of the monocular shapes also produces intuitive indices indicative of the laterality and type of ocular motility disorders. Comparison of the right eye’s shape to that of the left eye’s shape produces metrics indicative of binocular imbalances in synergistic muscle pairs (e.g., the right lateral rectus and left medial rectus), whereas comparison of the right eye’s shape to that of the left eye’s shape reflected about the vertical axis produces metrics indicative of binocular imbalances in the same muscle (i.e., the right and left lateral rectus). We set benchmarks to evaluate the ability of our image processing and statistical framework to capture normative patterns of non-strabismic ductions, shown in Figure 3 (Lim et al., 2014a). These benchmarks offer a way to assess the validity of these two components, as previous studies have described individual ductions but have not provided a statistical summary of their patterns (Lim et al., 2014a; Lee et al., 2019; Lou et al., 2022).

### 2.6 Boundaries

Benchmark III: duction magnitudes are anisotropic.

The derivation of all indices begins with treating a participant’s ductions as a collection of eight vectors $\vec{d\;}$ with magnitudes and directions $\left(d_{MAG}@d_{DIR}\right)$ and with origins of $\left(0,0\right)$ representing primary gaze. Each vector’s direction and magnitude are derived from the rotational axes ( n) and angles ( θ ) identified during optimization and are expressed in degrees. Two general operations are then performed on each eye’s collection of ductions: integration within the boundaries enclosed by the ductions is used to derive areas representative of movement extent and averaging of a participant’s duction vectors is performed to derive vector averages indicative of symmetry. The right- and left-eye’s indices are denoted by OD (oculus dexter) and OS (oculus sinister) subscripts.

### 2.7 Area

Benchmark IV: the right and left motor fields are equivalent.

Benchmark V: the motor discrepancy magnitude is smaller than the gaze discrepancy magnitude.

Our indices of area ask, “what is each eye’s total range of motion” and “are they the same?” The motor fields ( $MF_{OD}$ and $MF_{\mathrm{OS}})$ , first computed by Rowe and Hanif (2011), are the intraocular areas enclosed by all ductions. We chose this name to compliment the terminology expressing the total extent of sensory space (i.e., the visual field). The similarity of the motor fields is captured by the motor and gaze discrepancies ( MDisc and GDisc ) which compare the extent of “matching ductions” (e.g., adduction, abduction, elevation…) or matching gazes (e.g., leftward, rightward, upward…) between the eyes.

The motor fields are derived by drawing a border around the tips of each participant’s duction vectors (Eq. 6). This creates an enclosed polygon whose area is the raw motor field (Eq. 7). MATLAB’s polyshape and area functions were used for each respective step. We then express the motor fields relative to the group mean to avoid the use of non-intuitive units (i.e., degrees squared) and to center the values about one (Eq. 8). Evaluation of the motor field then allows one to determine whether the extent of motor space is less than or greater than the population average. The motor and gaze discrepancies are derived by superimposing the motor fields and identifying the relative proportion of non-overlapping (i.e., monocular) areas. In practice, this is accomplished with set theory operations which first take the difference between the motor field polygons and then divide the sum of monocular areas by the combined area of both motor fields. The left eye’s polygon is reflected about the vertical axis prior to computation of the motor discrepancy to allow the comparison of matching ductions (Eqs 9, 10). The gaze discrepancy instead compares the motor fields “as they are” (Eq. 11). The motor and gaze discrepancy values are thus bounded between zero and one: a value of zero is obtained when there is complete overlap between the superimposed motor fields (no discrepancy) whereas a value of one is obtained when there is no overlap between the superimposed motor fields (maximum discrepancy).


(6) $$\mathrm{shape}=\mathrm{polyshape}\left({\vec{d}}_{1\to n}\right)$$

(7) $$MF_{\mathrm{RAW}}=\mathrm{area}\left(\mathrm{shape}\right)$$

(8) $$MF=\frac{MF_{i}}{\mathrm{mean}\left(MF_{1\to N}\right)}$$

(9) $$\mathrm{shap}e_{\mathrm{OS}-\mathrm{REFLECTED}}=\left(x_{\mathrm{OS}}*-1,y_{\mathrm{OS}}\right)$$

(10) $$\begin{array}{l} \mathrm{MDisc}=\\ \;\frac{\begin{array}{l} {}[\left(\mathrm{shap}e_{OD}-\mathrm{shap}e_{\mathrm{OS}-\mathrm{REFLECTED}}\right)\cup \\ \left(\mathrm{shap}e_{\mathrm{OS}-\mathrm{REFLECTED}}-\mathrm{shap}e_{OD}\right)] \end{array}}{\sum \left[\mathrm{area}\left(\mathrm{shap}e_{OD}\right),\;\mathrm{area}\left(\mathrm{shap}e_{\mathrm{OS}-\mathrm{REFLECTED}}\right)\right]} \end{array}$$

(11) $$\mathrm{GDisc}=\frac{\left[\left(\mathrm{shap}e_{OD}-\mathrm{shap}e_{\mathrm{OS}}\right)\cup \left(\mathrm{shap}e_{\mathrm{OS}}-\mathrm{shap}e_{OD}\right)\right]}{\sum \left[\mathrm{area}\left(\mathrm{shap}e_{OD}\right),\mathrm{area}\left(\mathrm{shap}e_{\mathrm{OS}}\right)\right]}$$

The combination of motor fields and discrepancy values are informative of both normal and abnormal ocular-motor function. First, the motor fields of non-strabismic participants should be equivalent because the total magnitude of ductions is similar in each eye. Next, because the magnitude of matching ductions (i.e., adduction in the right-eye vs. adduction in the left-eye) are more similar than the magnitude of matching gazes (i.e., leftward in the right-eye vs. leftward in the left-eye), the magnitude of the motor discrepancy should be smaller than that of the gaze discrepancy. This is schematically illustrated in Figures 3C,D. The amount of “non-overlap” is minimal when comparing the shape of the right eye’s ductions to the shape of the left eye’s reflected ductions (the motor discrepancy) whereas the amount of “non-overlap” is larger when computing the gaze discrepancy. Regarding motility defects, because unilateral defects will always produce a smaller and differently shaped motor field in one eye, they are characterized by the presence of motor field asymmetry and resultantly large motor and gaze discrepancies. The opposite is true for some types of bilateral defects in which equivalent motor field reductions may present with either a large motor [e.g., gaze palsies (Strupp et al., 2014)] or gaze [e.g., vergence palsies (Ohtsuka et al., 2002)] discrepancy.

### 2.8 Symmetry

Benchmark VI: the muscle bias vectors should be equivalent in magnitude but opposing in direction.

Benchmark VII: the magnitude of the muscle difference vector should be smaller than the magnitude of the gaze difference vector.

Our indices of symmetry ask, “in which directions are ductions smallest and largest” and “in which directions are interocular differences smallest and largest?” Each index consists of two vectors: one pointing towards the largest value (pro) and the other pointing towards the smallest value (anti). Each of the symmetry indices can thus be considered dipoles which show the meridian along which duction magnitudes or their interocular differences vary most. The muscle biases ( ${\vec{MB}}_{OD}$ and ${\vec{MB}}_{\mathrm{OS}}$ ) show the meridian along which duction variability is greatest. Ductions are the result of forces exerted by multiple extraocular muscles, so in this spirit, the name “muscle bias” refers to the directions in which the collective forces are smallest or greatest (Kushner, 2006). The muscle difference ( $\vec{\mathrm{MDiff}}$ ) and gaze difference ( $\vec{\mathrm{GDiff}}$ ) instead show the meridian along which the magnitude of matching ductions or matching gazes vary most.

The muscle bias vectors are computed by first normalizing a participant’s ductions $\vec{d\;}$ : all vectors are divided by the magnitude of the largest vector (Eq. 12). This set of vectors, ${\vec{d\;}}_{N}$ , has a range of magnitudes which lie between zero and one. Second, the vector average of a participant’s normalized vectors is computed: this is the pro-muscle bias ${\vec{MB}}_{\mathrm{PRO}}$ (Eq. 13). Multiplying the components of ${\vec{MB}}_{\mathrm{PRO}}$ by −1.0 produces the opposing anti-muscle bias vector ${\vec{MB}}_{\mathrm{ANTI}}$ (Eq. 14). The initial normalization highlights intraparticipant duction asymmetries independent of the absolute duction magnitudes. The magnitude and direction of the muscle bias vectors are uniquely informative. The magnitude of the vectors, bounded between zero and one, represents the relative degree of intraocular asymmetry. The lower bound of zero occurs when a participant’s ductions are equivalent in magnitude and perfectly opposing in direction (minimal bias) and the upper bound of one occurs when a participant’s ductions are all executed in the same direction (maximal bias). The direction of the pro-muscle bias shows where duction magnitudes are typically largest whereas the direction of the anti-muscle bias shows where duction magnitudes are typically smallest.


(12) $${\vec{d\;}}_{N}=\frac{\vec{d}}{\max\left(∥\vec{d}∥\right)}$$

(13) $${\vec{MB}}_{\mathrm{PRO}}=\left({\vec{d}}_{N_{1}},{\vec{d}}_{N_{2}},\ldots {\vec{d}}_{N_{n}}\right)/n$$

(14) $${\vec{MB}}_{\mathrm{ANTI}}=-1*{\vec{MB}}_{\mathrm{PRO}}$$

The muscle difference and gaze difference vectors are also derived via vector averaging. The distinction is that now vectors from each eye ( ${\vec{d\;}}_{OD},{\vec{d\;}}_{\mathrm{OS}}$ ) are compared to each other. There are several steps for each computation. First, for the muscle difference, the left eye’s vectors are reflected about the y-axis to allow comparison of matching ductions (Eq. 15). Second, vector subtraction is performed: the absolute difference between vectors is taken and then assigned the sign of the right eye’s components. This creates a set of difference vectors, ${\vec{\mathrm{diff}}}_{\mathrm{MUSCLE}}$ , whose magnitudes represent the absolute differences in matching duction magnitude and whose directions maintain the right eye’s conventions (Eq. 16). The difference vectors are then normalized by dividing by the largest difference vector magnitude (Eq. 17). Like the muscle biases, this normalization highlights differences in duction differences independent of their absolute size. The pro-muscle difference is then computed by taking the vector average of the difference vectors, and the anti-muscle difference is formed by multiplying the pro-muscle difference components by −1.0 (Eqs 18, 19). The computation of the gaze difference vectors is equivalent to that of the muscle difference vectors except the left eye’s collection of vectors are not reflected about the y-axis prior to interocular comparison (Eq. 20). Then, after normalizing the difference vectors (Eq. 21), vector averaging is performed to compute the pro-gaze difference and the anti-gaze difference (Eqs 22, 23). The utility of the gaze difference vectors lies in their correlation to experiential measurements and functional adaptations. This is because diplopia should be most manifest in the direction where interocular differences in matching gazes are largest ( ${\vec{GD}}_{\mathrm{PRO}}$ ) and least manifest where interocular differences between matching gazes are smallest ( ${\vec{GD}}_{\mathrm{ANTI}}$ ). The gaze difference vectors thus reveal the direction in which habitual head turns may be adopted and the base direction in which prism may be prescribed to minimize symptoms ( ${\vec{GD}}_{\mathrm{PRO}}$ ).


(15) $${\vec{d\;}}_{OS_{\mathrm{REFLECTED}}}=\left[d_{OS_{X}}*-1,d_{OS_{Y}}\right]$$

(16) $$\begin{array}{l} {\vec{\mathrm{diff}}}_{\mathrm{MUSCLE}}=[\mathrm{abs}\left({d_{OD}}_{X}-{d_{\mathrm{OS}}}_{X_{\mathrm{REFLECTED}}}\right)*\mathrm{sign}\left({d_{OD}}_{X}\right),\;\\ \;\mathrm{abs}\left({d_{OD}}_{Y}-{d_{\mathrm{OS}}}_{Y_{\mathrm{REFLECTED}}}\right)*\mathrm{sign}\left({d_{OD}}_{Y}\right)] \end{array}$$

(17) $${\vec{\mathrm{diff}}}_{\mathrm{MUSCL}E_{N}}=\frac{{\vec{\mathrm{diff}}}_{\mathrm{MUSCLE}}}{\max\left(∥{\vec{di\mathrm{ff}}}_{\mathrm{MUSCLE}}∥\right)}$$

(18) $${\vec{\mathrm{MDiff}}}_{\mathrm{PRO}}=\left({\vec{\mathrm{diff}}}_{\mathrm{MUSCL}E_{1}},\ldots {\vec{\mathrm{diff}}}_{\mathrm{MUSCL}E_{n}}\right)/n$$

(19) $${\vec{\mathrm{MDiff}}}_{\mathrm{ANTI}}=-1*{\vec{MD}}_{\mathrm{PRO}}$$

(20) $$\begin{array}{l} {\vec{\mathrm{diff}}}_{\mathrm{GAZE}}=[\mathrm{abs}\left({d_{OD}}_{X}-{d_{\mathrm{OS}}}_{X}\right)*\mathrm{sign}\left({d_{OD}}_{X}\right),\\ \;\mathrm{abs}\left({d_{OD}}_{Y}-{d_{\mathrm{OS}}}_{Y}\right)*\mathrm{sign}\left({d_{OD}}_{Y}\right)] \end{array}$$

(21) $${\vec{\mathrm{diff}}}_{GAZE_{N}}=\frac{{\vec{\mathrm{diff}}}_{\mathrm{GAZE}}}{\max\left(∥{\vec{\mathrm{diff}}}_{\mathrm{GAZE}}∥\right)}$$

(22) $${\vec{\mathrm{GDiff}}}_{\mathrm{PRO}}=\left({\vec{\mathrm{diff}}}_{GAZE_{1}},\ldots {\vec{\mathrm{diff}}}_{GAZE_{n}}\right)/n$$

(23) $${\vec{\mathrm{GDiff}}}_{\mathrm{ANTI}}=-1*{\vec{GD}}_{\mathrm{PRO}}$$

The indices of symmetry are further informative regarding the spectrum of ocular-motor function. The anisotropic ductions of non-strabismic participants should produce muscle biases equivalent in magnitude but opposing in direction because the largest ductions, typically depression and adduction, are equivalent between the eyes but oriented oppositely about the vertical axis. Thus, as with the motor fields and discrepancies, one would expect to find muscle difference vectors with magnitudes less than that of the gaze difference vectors because matching ductions are more similar in magnitude than that of matching gazes. The values of the motor fields and discrepancies can indicate abnormal ductions; however, do not show “where” the problem lies. This is accomplished by the indices of symmetry which “point towards” the affected directions. For example, coincidence of the muscle bias, muscle difference, and gaze difference directions is strongly suggestive of movement limitation in a single direction because the direction of the smallest duction (anti-muscle bias) is the direction where interocular differences are largest (pro-muscle and pro-gaze differences). The coincidence of all vector magnitudes is instead suggestive of movement limitation in opposing directions because the lack of intraocular or interocular asymmetry results in muscle bias, muscle difference, and gaze difference vectors with magnitudes near zero. For bilateral palsies, comparison of each eye’s muscle bias vectors can also impart differentiation between motility defects: the individual muscle bias directions will be either coincident or opposing when the same or different movement directions are impacted in each eye. In both cases, small magnitude difference vectors support the diagnosis of gaze and/or muscle palsies: there is a difference in movement extent that is symmetric about primary gaze.

### 2.9 Cross-sectional and longitudinal difference criteria

The initial two steps of our duction recording protocol produced estimates of a participant’s individual ductions as well as their overall area and symmetry. This data can be used for cross-sectional analyses which compare an individual to a normative population. How does a clinician next determine if these quantifications are indicative of longitudinal changes (i.e., pathological progression or therapeutic improvement)? The final component of our recording protocol sets this standard using a statistic called the minimal detectable change (MDC). The MDC, also known as the within-subject standard deviation, represents the extent by which a value must change to surpass the anticipated magnitude of measurement error (Beekhuizen et al., 2009; Negrete et al., 2010; Furlan and Sterr, 2018; Piedrahita-Alonso et al., 2022). It is expressed in the unit of measurement (e.g., for ductions, in degrees, and for indices, arbitrary units) and is derived from the standard error of measurement (Eq. 24) (Piedrahita-Alonso et al., 2022). The MDC is preferable to other statistics [e.g., coefficients of repeatability (Dolman et al., 2012)] because it is not biased by the magnitude of interparticipant variability (Piedrahita-Alonso et al., 2022).


(24) $$MDC_{95}=1.96*SEm*\sqrt{2}$$

The z-score of 1.96 utilized in our calculation establishes a 95% confidence interval for the anticipated differences, while the radical term at the end accounts for the uncertainty associated with repeated measurements.

The objective of our final set of computations was to compute a distribution of MDC values for individual ductions, indices of area, and indices of symmetry. These computations establish the magnitude by which a participant’s ductions, movement area, or movement symmetry must change to exceed fluctuations expected from measurement error. To compute the range of possible MDC values for each metric, we performed multiple iterations of repeated measures ANOVAs on subsets of randomly selected data. This approach produces the standard error of measurement, necessary for computation of the MDC, via the repeated measures ANOVA’s mean squared error (Eq. 25) (Atkinson and Nevill, 1998).


(25) SEm=MSE=SSEdf

For duction magnitudes, we created 20 × 2 matrices holding two randomly chosen magnitudes selected from each of the twenty participant’s collection of ductions. This was done for each duction and eye; therefore, there were a total of sixteen 20 × 2 matrices each holding two out of the possible three least-error duction magnitudes within each duction and eye combination. A repeated measures ANOVA was run on each matrix to derive the mean squared error, standard error of measurement, and thus a single MDC value. The order of each participant’s magnitudes and directions were next shuffled and the preceding steps repeated 1,000 times to create a full distribution of possible MDC values. A similar approach was used to compute the MDC values for duction directions and for each index. For the indices of area and symmetry, the 20 × 2 matrices were formed by randomly selecting one rotation estimate from each of the eight ductions to compute each respective index. One more random selection was then performed to create a 20 × 2 matrix containing two estimates of each index for each participant. A repeated measures ANOVA was then run, and random selection repeated, until 1,000 estimates of the MDC for each index had been computed. Shuffling was performed to ensure that most sequential combinations within and across participants were sampled. We did not set a benchmark for this section of our protocol, as MDC values have not yet been established for individual ductions. Furthermore, neither normative ranges nor MDC values have yet been defined for motility patterns. For a flow chart schematizing the entire protocol, please see Supplementary Figure S2.

## 3 Results

### 3.1 Image processing

**Figure 4:**
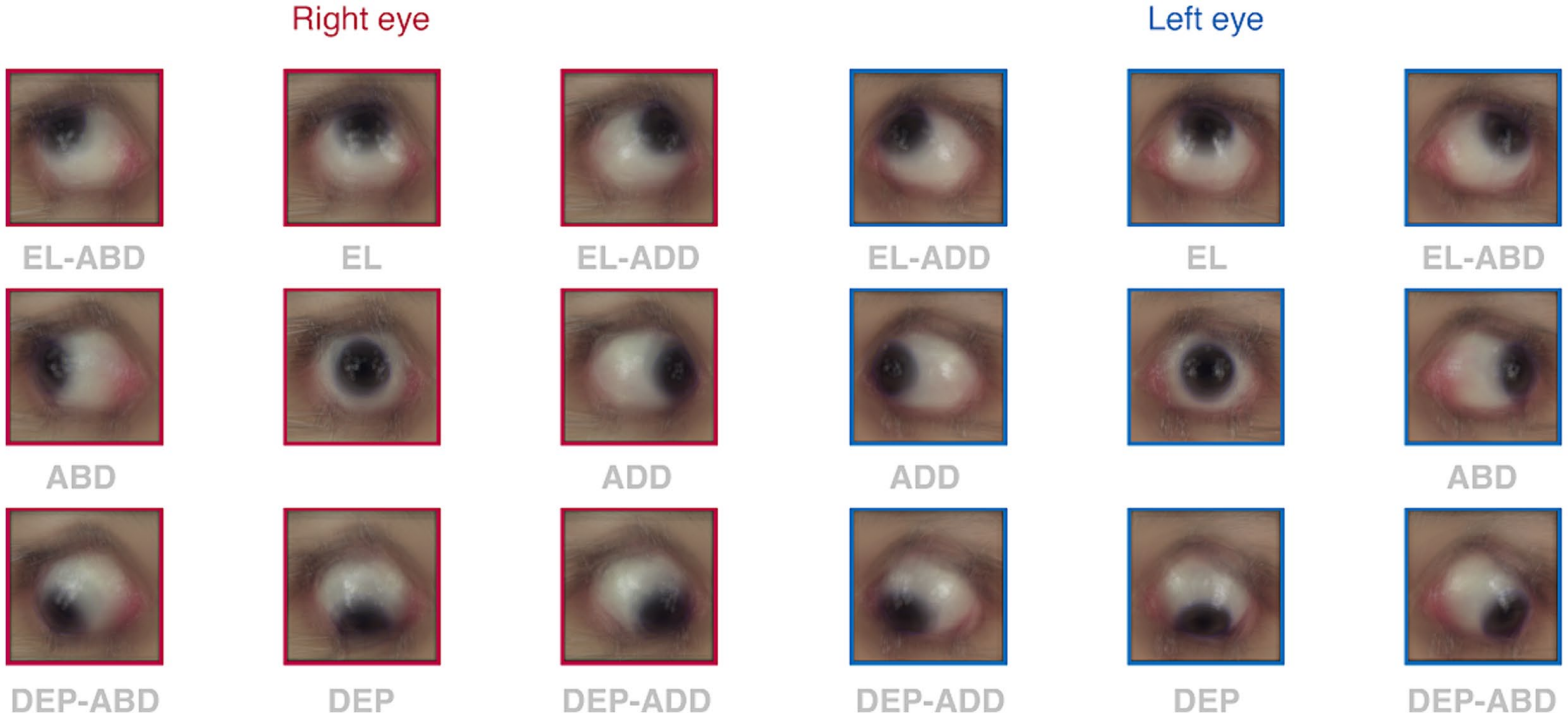
Average images captured for each duction. Each square shows the group mean photo obtained after averaging within and then across participants for all ductions in each eye (red borders = right eye, blue borders = left eye). Annotations show the gaze in which the eyes were directed.

Figure 4 shows the average input to our image processing pipeline: a collection of one-thousand images collected in primary (1 frame from each eye) and eccentric gazes (3 frames from each of the 8 ductions and eyes) from twenty participants. Our benchmark of gaze-independent model metrics was nearly achieved. Our mask R-CNN’s overall metrics of accuracy (98.94%), sensitivity (94.50%), specificity (99.31%), and precision (91.93%) show that it successfully segmented the iris, and thus limbal boundaries, of our participants (see Eqs 1–4). The sensitivity of the mask R-CNN varied slightly across gazes: it was highest in upward (98%) and lowest in downward (86%) gaze positions. The precision was similarly variable: it was highest for ductions executed down and right (95%) and lowest for those executed straight down (89%). This shows that downward movements had the highest number of false negatives (missed pixels) and false positives (misidentified pixels), respectively. An analysis of mean pixel locations in each category showed that this is because the model tended to underestimate the vertical location of limbus boundaries for downward movements: the mean vertical location of missed pixels was 260.11 (closer to the bottom of image) and that of mismarked pixels was 251.89 (closer to primary gaze). We used the average pixel to millimeter conversion to compute that this ten-pixel difference could account for approximately 0.70° of underestimation. The predominance of these mislabelings suggests that future iterations of the model may need to be exposed to, or “trained on,” a more extensive set of gaze downward photos.

There was also evidence of limited gaze-dependence in our error computations. The group mean error of the selected rotations (OD: 0.35 mm, OS: 0.40 mm) was significantly smaller than that of the discarded rotations (OD: 0.46 mm, OS: 0.51 mm) in each eye (*p* < 0.001), confirming that the selection process did indeed remove estimates with the most error. The error between observed and estimated limbus boundaries was largest in adduction for both eyes (OD: 0.52 mm, OS: 0.69 mm). A comparison of the observed and estimated coordinates for adductive movements showed that the magnitude of adduction was likely underestimated in each eye. This is because the estimated ellipses for adductive movements were shifted in the abductive direction in each eye [i.e., rightward (0.21 @ 171.63°) and leftward (0.37 @ 0.54°) in the right and left eyes, respectively]. This matched a general trend for estimated ellipses to be displaced more rightward in the right eye and more leftward in the left eye when compared to the marked boundaries. The amount of potential error induced by these discrepancies was slightly larger than of that imposed by segmentation inaccuracies (~ 3° in both eyes).

### 3.2 Cross-sectional and longitudinal differences in duction patterns

Our protocol captured the interparticipant variability and anisotropy characteristic of ocular ductions. This is shown by the group mean magnitudes and directions of the individual ductions, the group mean area indices, and the group mean symmetry indices. We show in the following sections that the latter two groups of data are convenient ways of summarizing multiple movements into singular metrics.

**Figure 5:**
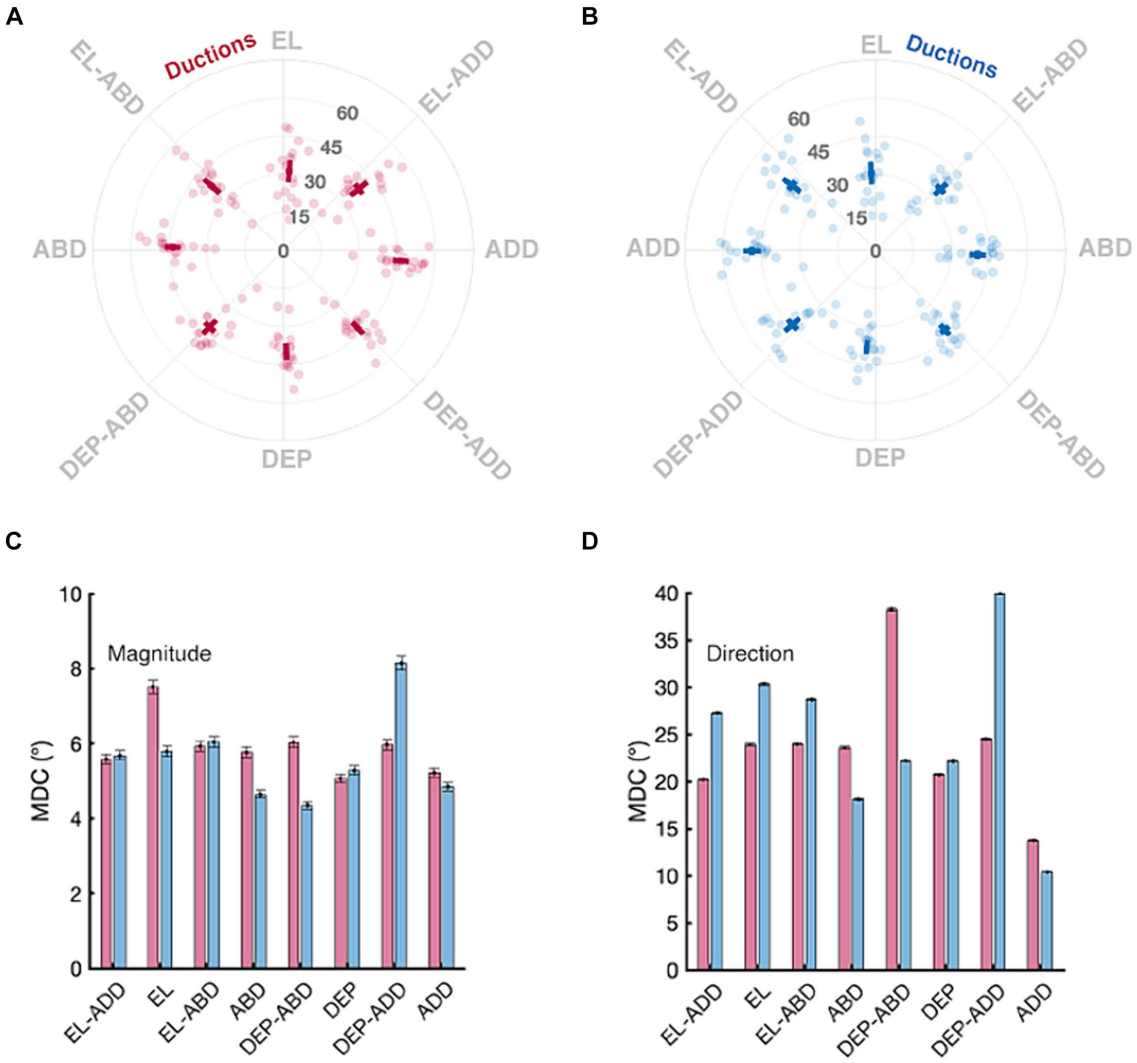
Normative and minimal detectable change (MDC) values for duction magnitude and direction. Panels in the top row show the mean magnitude and directions (solid square) with confidence intervals (solid lines, +/− 1.96 SEM) for the right **(A)** and left **(B)** eyes. Data from individual participants are shown as transparent circles. The magnitude is denoted by the grey numbers (degrees) and the direction, in increments, by the labels on the circumference of each panel. Panels in the bottom row show the MDC values for each duction’s magnitude **(C)** and direction **(D)**; the error bars are confidence intervals representing the mean +/− 1.96 SEM MDC values derived from sequential resampling.

The top row of Figure 5 shows the distribution of duction magnitudes and directions for all participants. Duction magnitudes ranged between a minimum of 11.25° to a maximum of 62.87° across all directions, eyes, and participants. Participants with the overall smallest and largest ductions had mean magnitudes of 24.82° and 49.46° across all directions and both eyes, respectively, with the mean duction magnitude being equivalent between the eyes for all participants (95% CI OD: 38.65–41.82°, OS: 38.70–41.36°, *p* = 0.75). The mean direction of each duction was consistent with the instructed meridians. Adductive (95% CI OD: 45.00–48.31°, OS: 46.90–50.50°) and adductive-depressive (95% CI OD: 40.78–44.01, OS: 41.87–45.84°) movements tended to be the largest for both eyes. Each eye’s ability to move upward was smallest. (95% CI OD: 29.03–33.58°, OS: 28.18–32.69°). This amount of normative variability in duction magnitude across participants and duction directions matches what has been previously documented with both image processing (Lim et al., 2014a; Lee et al., 2019; Lou et al., 2022) and psychophysical techniques (Shechtman et al., 2005). The bottom row of Figure 5 shows that the MDC values we computed for duction magnitude were instead remarkably consistent across ductions and tended to have values ranging between 4° and 8°. This means that our participants tended to repeatedly execute similarly sized ductions and that our protocol was able to measure each direction with similar precision. This range sets a threshold for signaling clinically significant longitudinal change: if a participant’s individual movement extent changes by >4°, further investigation is warranted. The MDC values for duction direction, albeit larger, can similarly be used to detect longitudinal changes in movement direction.

**Figure 6:**
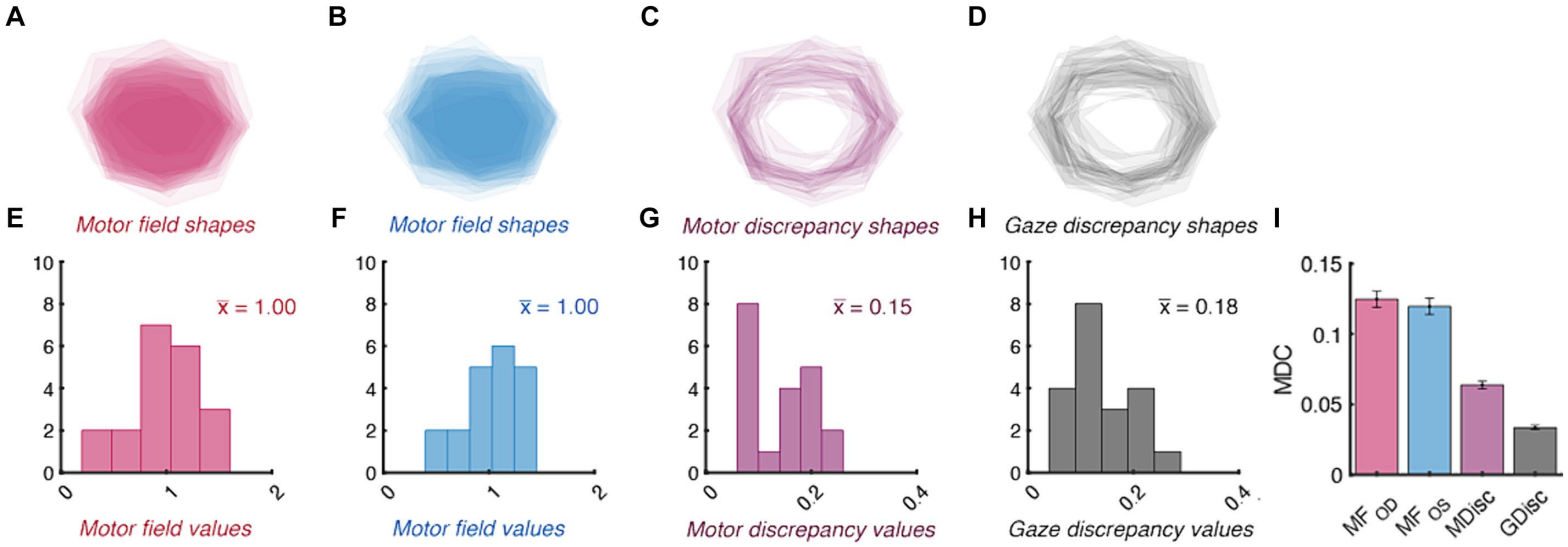
Normative and minimal detectable change (MDC) values for duction area. Top panels show the average shape of each participant’s right- **(A)** and left-eye **(B)** motor fields, motor discrepancies **(C)**, and gaze discrepancies **(D)**. The individual shapes are set to have a transparency of 1 / number of participants; therefore, the relative opacity of each shape shows regions common to a small (translucent) vs. large (opaque) number of participants. The bottom panels show the distribution of motor field **(E,F)**, motor discrepancy **(G)**, gaze discrepancy **(H)**, and MDC values **(I)** for each index of area. The group mean value for each index is indicated with colored text in panels **(E–G)**. The error bars in Panel **(I)** are confidence intervals representing the mean +/− 1.96 SEM MDC values derived from sequential resampling.

The area encompassed by the motor fields and the total area of discrepancy between them also conformed to our *a-priori* benchmarks (Figure 6). Each eye’s total movement extent was similar: the raw motor fields were not significantly different when compared between the right- and left-eyes (95% CI $MF_{RAW_{OD}}$ : 4309.15–4973.65 deg. squared, $MF_{RAW_{\mathrm{OS}}}$ : 4253.88–4822.28 deg. squared, *p* = 0.47). Figure 6 shows that, when normalized, our population’s right and left motor fields spanned values between ~15% smaller and larger than the group average (95% CI $MF_{OD}$ : 0.86–1.14, $MF_{\mathrm{OS}}$ : 0.88–1.12). This is shown by the “halo” of translucent shading in the peripheral regions of the motor fields in Figures 6A,B. The distribution of motor discrepancy values was not significantly smaller than the gaze discrepancies as expected: their confidence intervals overlapped (95% CI MDisc : 0.12–0.17, GDisc : 0.11–0.16). Their similarity is also shown by the average shapes via which each index was derived: the motor and gaze discrepancy shapes all resembled thin lines traversing high magnitude regions of the motor fields. The amount by which each of the area metrics need to change for clinical significance is shown in Panel I. Importantly, because each of the MDC values is smaller than that of the confidence intervals (and thus the normative ranges), it is likely that our protocol could detect baseline deviations in total movement area prior to these metrics becoming abnormal.

**Figure 7:**
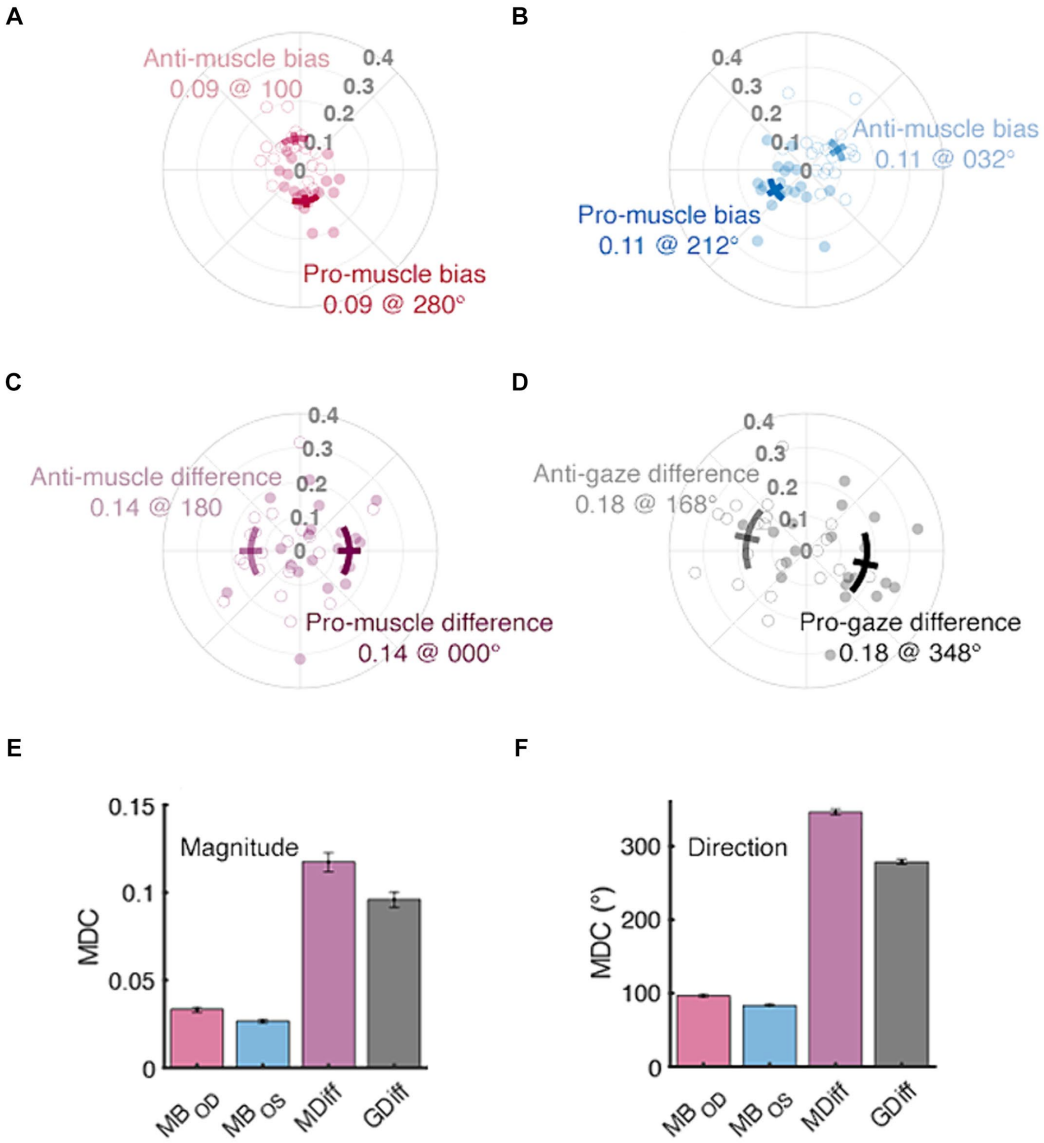
Normative and minimal detectable change (MDC) values for duction symmetry. The distributions of muscle bias, muscle difference, and gaze difference vectors are shown in panels **(A–D)**, respectively. Filled vs. unfilled circles are data from individual participants’ pro- and anti-vectors, respectively. Confidence intervals (solid lines, +/− 1.96 SEM) are shown as either solid (pro) or translucent (anti) arcs and further differentiated by adjacent text containing labels and respective group mean values. The bottom row’s panels contain MDC values for each index’s magnitude **(E)** and direction **(F)**; the error bars are confidence intervals representing the mean +/− 1.96 SEM MDC values derived from sequential resampling.

The muscle bias and difference vectors bring forward the anisotropy noted in individual ductions (Figure 7). The pro-muscle bias vectors tended to point inward and downward, thereby highlighting the relative “strength” of adductive and downward movements in each eye (Figure 7A, 95% CI ${\vec{MB}}_{PRO_{OD}}$ : 0.08–0.10 @ 255.70–304.15°, Figure 7B, ${\vec{MB}}_{PRO_{\mathrm{OS}}}$ : 0.10–0.12 @ 193.89° - 231.11°). This simultaneously highlights the relative “weakness” of abductive and upward movements (95% CI ${\vec{MB}}_{\mathrm{ANT}I_{OD}}$ : 0.08–0.10 @ 75.70–124.15°, ${\vec{MB}}_{\mathrm{ANT}I_{\mathrm{OS}}}$ : 0.10–0.12 @ 13.89° - 51.11°). The average difference between a participant’s right vs. left muscle bias directions was close to the 90° expected by the approximate mirror symmetry of the extraocular muscles (95% CI: 62.52–77.43°), thus meeting our benchmark of opposing muscle bias directions. Further, because the muscle bias magnitudes were equivalent in size (*p* = 0.20), these findings show that the asymmetry in extraocular muscle function was similar between the eyes. The pro- and anti-muscle difference vectors (Figure 7C, 95% CI ${\vec{\mathrm{MDiff}}}_{\mathrm{PRO}}$ : 0.13–0.16 @ 331.83–28.60°, ${\vec{\mathrm{MDiff}}}_{\mathrm{ANTI}}$ : 0.13–0.16 @ 151.83° - 208.60°) show that adductive vs. abductive movements tended to be the most vs. least different when compared between a participant’s eyes. The variability in adduction magnitude has been noted previously and may be attributable to occlusion of the opposing visual field one’s nose (Clark and Isenberg, 2001). The group mean pro- and anti-gaze difference vectors told a similar story: leftward vs. rightward movements were most vs. least different following interocular comparison (Figure 7D, 95% CI ${\vec{\mathrm{GDiff}}}_{\mathrm{PRO}}$ : 0.14–0.21 @ 318.18–17.13°, ${\vec{\mathrm{GDiff}}}_{\mathrm{ANTI}}$ : 0.14–0.21 @ 138.18° - 197.13°). This suggests that a participant’s ability to adduct with their right-eye or abduct with their left-eye may have been most variable during data collection. The gaze difference vectors did have slightly larger mean magnitudes (0.18 vs. 0.14); however, were not significantly different when compared to the muscle bias vectors (*p* = 0.06). This is in line with the equivocal comparisons found in our measures of area and again suggests that the normal difference between matching ductions and matching gazes may be small in non-strabismic participants. The final two panels of Figure 7 show the MDC values for our symmetry measures segregated by vector magnitude and direction. Figure 7F shows that our protocol is least sensitive to changes in the mean direction of asymmetry. First, because the muscle biases would need to change by at least ~90° to be longitudinally detected, small changes in asymmetry could be missed. Similarly, the >180° values computed for the muscle difference and gaze difference vectors suggest that the derivation of these vector directions was highly sensitive to the individual ductions used to compute them. Reduction of the MDC values, and thus the measurement error inherent to our protocol, is explored in the Discussion section below.

## 4 Discussion

Our protocol is the first capable of automatically describing and detecting cross-sectional and longitudinal differences in the pattern of one’s ocular ductions. This was accomplished by integrating image acquisition, machine learning, mathematical optimization, summary statistics, and criterion setting. Comparison of the resultant image segmentations, rotation optimizations, area and symmetry descriptions, normative ranges, and change thresholds to our benchmarks establish the validity of our protocol and show that it can produce repeatable quantifications of a participant’s pattern of ductions.

The primary objective of this manuscript is to demonstrate that our proposed protocol extends the number of ways in which clinicians and scientists may analyze ocular motility disorders. Motility defects often present with stereotypical movement patterns, yet, there have been limited attempts to summarize and quantify them. This scarcity of numeric ocular motility data has left hypotheses regarding the etiology and natural history of many paralytic strabismic disorders largely untested. For example, it is unclear how compressive vs. ischemic lesions of cranial nerves differentially impact ocular motility. A retrospective case report showed that most pupil-sparing ischemic lesions imparted diffuse ocular motility defects whereas compressive lesions imparted focal defects (Sanders et al., 2001). If true, this differentiation could provide clarity to the still debated question of ordering costly neuroimaging in presumed ischemic nerve palsies (Chou et al., 2004; Volpe and Lee, 2014). Our framework has the power to make this differentiation because each pattern manifests as a different set of summary statistics. Unilateral diffuse motility defects are characterized by a small motor field and large discrepancies, whereas large magnitude muscle bias and difference vectors are instead the distinguishing feature of focal defects. The recovery phase of ocular pareses can also benefit from quantification. This is because the resolution of unilateral ocular pareses is typically judged using symptom-based criteria (Kim et al., 2018). Using the MDC to evaluate the evolution of a patient’s area or symmetry indices towards their pre-paresis values provides a firm criterion against which to judge recovery. Further, performing large-scale studies of both range of motion and eye movement dynamics throughout the recovery phase has the potential to make past intuitions about the natural history of pareses [e.g., “recovering muscles can transmit high, but not low, frequency discharges” (Wong et al., 2006), “ischemic palsies have the highest recovery rate,” “ischemic palsies resolve faster” (Kim et al., 2018)] concrete. Similar diagnostic and prognostic analyses are warranted for other conditions in which anecdotal evidence is used to “digest” variable motility presentations (e.g., thyroid ophthalmopathy (Salvi et al., 2015; Campi et al., 2021), myasthenia gravis (Cleary et al., 2008; Almog et al., 2016; de Meel et al., 2019), multiple sclerosis (Serra et al., 2018), and Miller-Fisher syndrome (Ryu et al., 2019)).

Objective motility measurements also have the potential to shed light on the natural history and etiology of non-paralytic strabismus. This is because the normal appearance of ductions in this patient population may have obscured subtle extraocular muscle asymmetries as causative factors. For example, it is not clear why current optometric measurements fail to predict which adults undergo heterophoric decompensation later in life (Jenkins et al., 1989). Similarly, although the presence of congenital exotropia and esotropia has long been assumed to abnormal brain circuitry (Quoc and Milleret, 2014); it is possible that the biomechanical properties of the extraocular muscles are instead a causative factor in strabismic development in these two groups (Moon et al., 2021). Quantification of duction area and symmetry in both groups can “crack open” both lines of investigation. The most exciting potential application of our protocol is to detail the efficacy of pharmacological-, surgical-, or therapy-based interventions via motility patterns in both paralytic and non-paralytic strabismus populations (Ciuffreda, 2002; Kapoor and Ciuffreda, 2002; Salvi et al., 2015; Özkan, 2016). This is critical to establish whether observation, surgery, vision therapy, or a combination of all three is optimal for recovery and maintenance of extraocular muscle function.

There are several minor shortcomings to address before our protocol is adopted. First, it is necessary to collect data in a larger segment of the non-strabismic population. The relatively small sample size used in the current investigation did not enable any of our indices to be stratified by demographics or biometrics. This is necessary to create a true normative database against which cross-sectional comparisons can be made. Second, we also plan on performing longitudinal data collection to determine if and how the indices of non-strabismic participants change over time. We compared a participant’s ductions and indices collected within a single recording session; however, it is also critical to establish our protocol’s repeatability over longer time scales before investigations of motility disorders commence. This is important because the ocular range of motion becomes smaller with age (Shechtman et al., 2005; Lee et al., 2019). Therefore, pathological changes in ductions, their area, or symmetry must first be differentiated from normal age-expected changes (e.g., reduction in elevation and thus smaller motor fields and a shift in muscle biases) for accurate diagnosis. Last, because ophthalmic speculums are uncomfortable, it may be more practical to have clinicians hold the patient’s eyelids while imaging is performed. We chose to use a speculum so that the position of the bullseye sticker was not obscured or moved by the clinicians fingers; however, because the amount of bite-bar stabilized head movements were small, sacrificing post-hoc correction for patient comfort may be a compromise worth making. Foregoing the semi-invasive nature of the bite bar may be a similar compromise worth making if clinicians deem a combined chinrest forehead rest capable of stabilizing the head.

The current experiment has laid a foundation for several future lines of research. We first plan on comparing the rotational estimates derived from our protocol to those derived from a video-based eye tracker to determine the relative accuracy and precision of our image processing pipeline. Similarly, comparing our protocol’s indices and MDC values to judgements issued by strabismologists can provide a complementary analysis which highlights the relative strengths of automated vs. subjective diagnoses. Placing our proposed image processing approach in line with either video-based trackers and/or expert clinicians necessitates including several minor but important modifications to our protocol in the future (e.g., stringent control of fixation using computerized displays, continued training to maximize segmentation accuracy, and quantitative comparison of optimization algorithms to minimize error). These modifications will reduce the measurement error and thus produce MDC values reflective of the true variability inherent to large ocular rotations. Second, we plan to continue creating and analyzing statistics which describe and capture changes in motility patterns. The indices laid out in this manuscript are a starting point from which development of others can proceed. For example, the rigid assumption that primary gaze is the location from which all ductions emanate can be alleviated by instead finding the centroid of each participant’s motor field, a practice which may need to be utilized in paralytic strabismics unable to move their eyes into primary gaze. Similarly, in addition to using vector averages, computing the minimum and maximum movement vectors may be more useful for capturing complex motility patterns. These computational explorations, in conjunction with accurate and precise measurement of strabismic eye movements, will identify the set of pattern descriptors and dynamic movement features most predictive of pathology and produce diagnostic criteria capable of sensing small deviations in ocular motility.

The “clinical eye” has long been used to ascertain a patient’s ocular-motor status. Recent and continuing calls to produce a valid and reliable estimate of ocular rotations have gradually shown that this tradition, while irreplaceable, cannot provide the accurate measurement, efficient documentation, and ease of communication imparted by standardized techniques (Lea and Gernardt, 1995; Dolman et al., 2012). Now, it is imperative to support the development of an image processing device incorporating our proposed techniques, in order to align the study of ocular motility with other advancements in eye care. As the integration of image processing and machine learning continue to shape clinical practice (Wang et al., 2023), it is important to embrace these technologies and use the information provided by them to determine if one’s eye movements differ from others or from their past selves.

## Data availability statement

The original contributions presented in the study are included in the article/Supplementary material, further inquiries can be directed to the corresponding author.

## Ethics statement

The studies involving humans were approved by the Institutional Review Board (IRB) at Nova Southeastern University. The studies were conducted in accordance with the local legislation and institutional requirements. The participants provided their written informed consent to participate in this study. Written informed consent was obtained from the individual(s) for the publication of any identifiable images or data included in this article.

## Author contributions

KW: Conceptualization, Formal analysis, Funding acquisition, Investigation, Methodology, Project administration, Writing – original draft. VC: Investigation, Project administration, Writing – review & editing. HR: Conceptualization, Software, Writing – review & editing.
