# Supplementary figures and images for: A protocol to quantify cross-sectional and longitudinal differences in duction patterns
Source: Front Neurosci. 2024 Jun 11;18:1324047. doi: 10.3389/fnins.2024.1324047 (PMC11196818; doi:10.3389/fnins.2024.1324047)

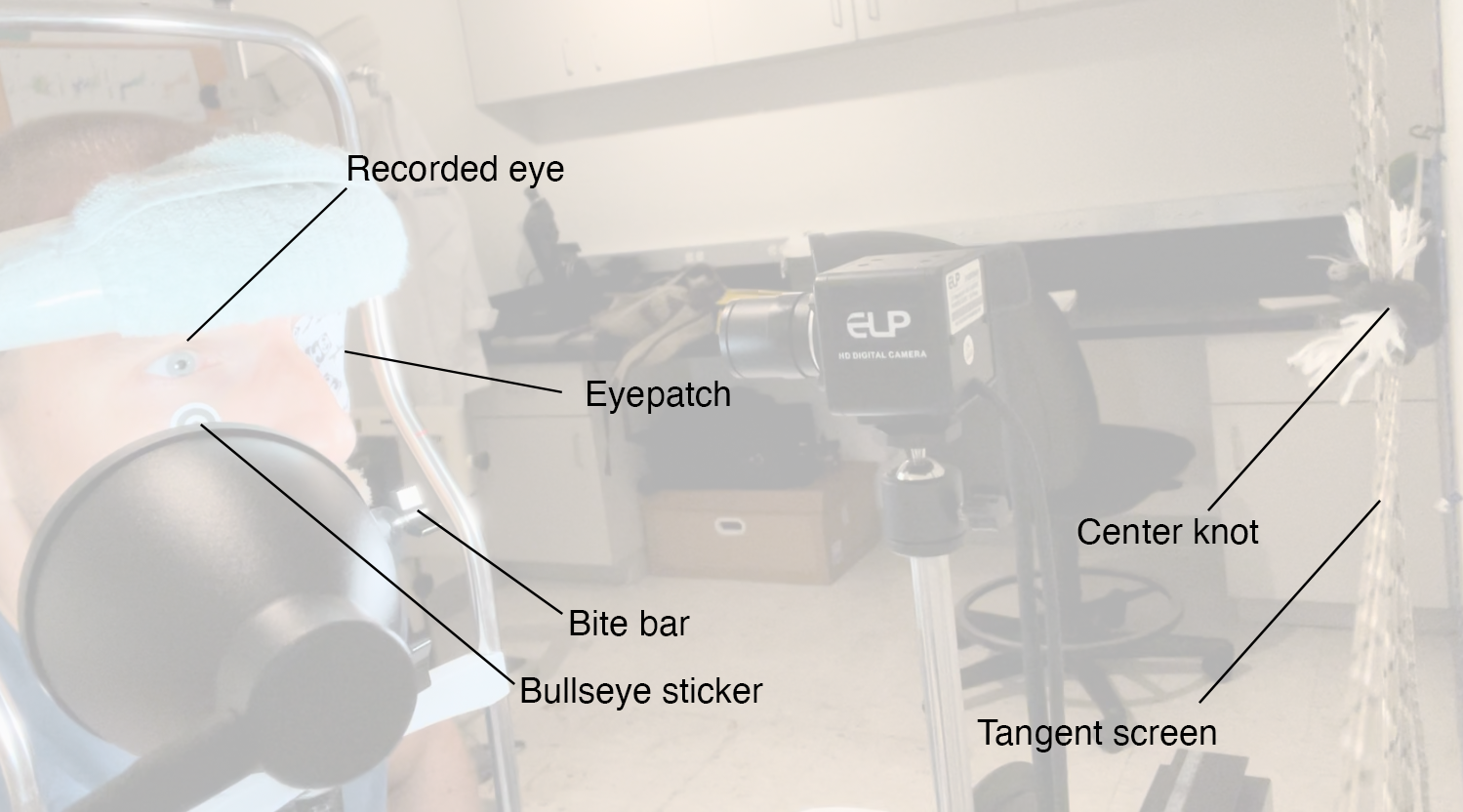

Supplement: Supplementary file 1 [file Image_1.TIF]

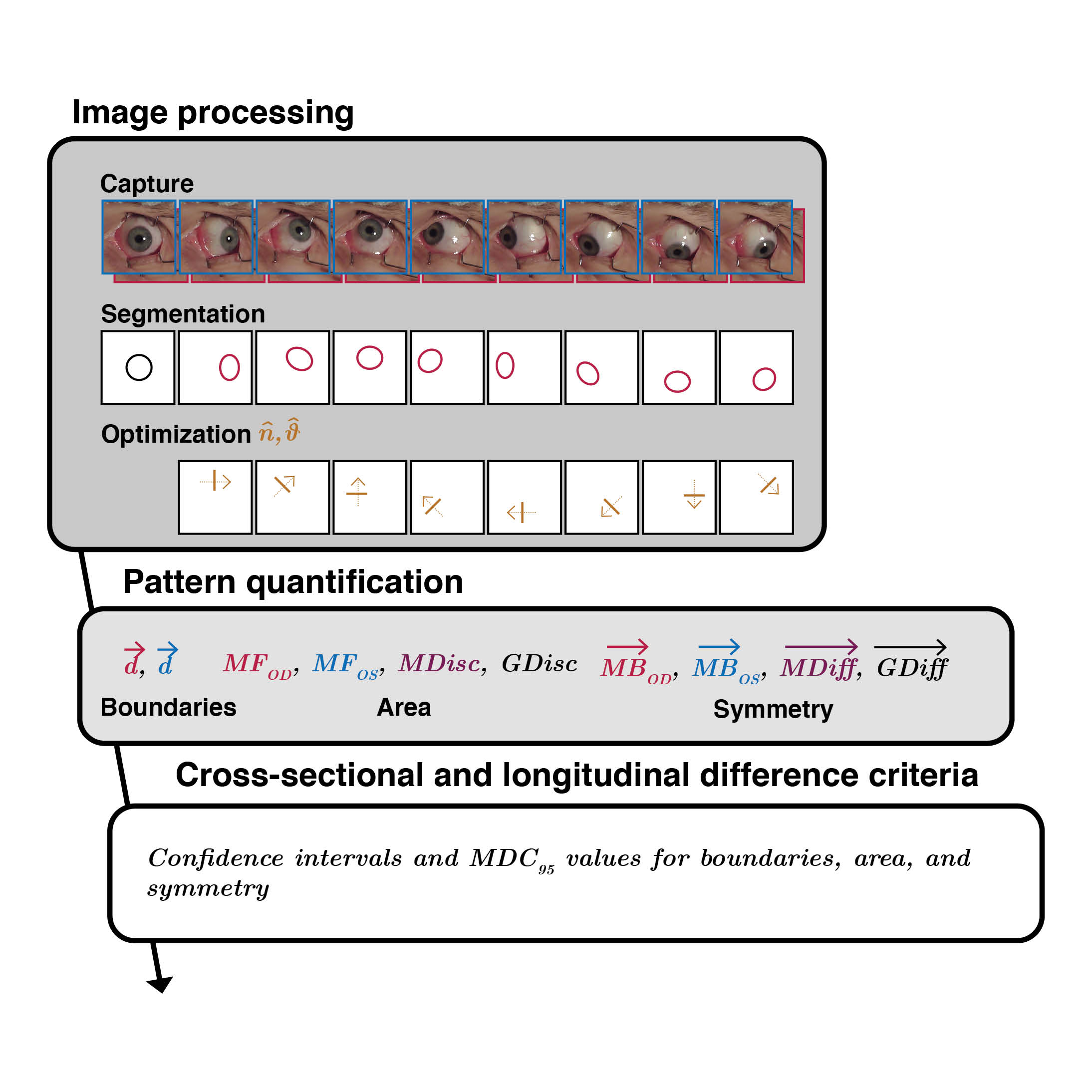

Supplement: Supplementary file 2 [file Image_2.JPEG]
